# Linking bacterial life-history strategies and diversity to litter decomposition dynamics in a dry-hot valley area

**DOI:** 10.3389/fmicb.2026.1766521

**Published:** 2026-02-06

**Authors:** Tao Yang, Chunjuan Shi, Enfu Chang, Yun Zhou, Pinrong Li, Qiang Liu, Xiqing Zhang, Jing Pang

**Affiliations:** 1 Yunnan Forestry Technological College, Kunming, Yunnan, China; 2 School of Biological Sciences and Technology, Beijing Forestry University, Beijing, China; 3 Yunnan Academy of Forestry and Grassland, Kunming, Yunnan, China; 4 Yunnan Key Laboratory of Biodiversity of Gaoligong Mountain, Kunming, Yunnan, China

**Keywords:** bacteria community, dry-hot valley, life-history strategies, litter chemical properties, litter decomposition

## Abstract

Litter decomposition is a critical ecosystem process that influences nutrient cycling and carbon sequestration, yet the role of microbial communities, especially bacteria, in driving decomposition dynamics is not well understood, particularly in stress-prone ecosystems. This study examines the relationship between bacterial life-history strategies, community diversity, and litter chemical properties during the decomposition of six herbaceous plant species in a dry-hot valley ecosystem. Over a 493-day period, we monitored litter mass loss, chemical composition (C, N, P, lignin, cellulose) at five decomposition stages (T1_69–T5_493), and bacterial community shifts at three representative stages (T1_69, T3_271, and T5_493). Our results show that bacterial traits, including life-history strategies, explained a larger proportion of variance in litter decomposition rates compared to chemical properties. Litter mass loss followed a clear “fast–slow” temporal pattern, and species-specific exponential decay parameters (k) indicated interspecific differences in decomposition rates. Bacterial communities shifted significantly in diversity and composition, with oligotrophic bacteria becoming dominant in later stages. The abundance of bacterial groups was closely correlated with litter traits like lignin and cellulose, but not with nitrogen or phosphorus ratios. Random forest analysis identified key bacterial biomarkers, whose abundance varied across decomposition stages, and canonical correspondence analysis emphasized the role of litter quality gradients (particularly cellulose and lignin) in shaping bacterial community structure. These findings highlight the importance of integrating microbial strategies and litter chemistry to understand decomposition dynamics, especially in water-limited ecosystems.

## 1 Introduction

Litter decomposition is a cornerstone of terrestrial ecosystem functioning, regulating the return of essential nutrients to the soil and playing a central role in global carbon cycling (Akoto et al., 2022; Zhao et al., 2025). Through the microbial breakdown of plant-derived organic matter, decomposition governs the supply of available nutrients that sustain plant productivity and microbial activity, while also facilitating the stabilization of soil organic matter and long-term carbon sequestration (Cotrufo et al., 2013; Bai et al., 2021). This process is especially critical in the context of climate change, as decomposition dynamics influence both atmospheric CO_2_ feedback and belowground nutrient retention. Despite its ecological significance, the mechanisms governing decomposition remain complex and variable across ecosystems, particularly in regions experiencing pronounced climatic or edaphic stress (Błońska et al., 2021; Babur et al., 2022).

Microorganisms, particularly fungi and bacteria, are the primary biotic agents driving litter decomposition (Buresova et al., 2021). Their roles extend beyond enzymatic degradation to include the regulation of nutrient stoichiometry, the modulation of soil microbial food webs, and the mediation of carbon and nitrogen transformations at various stages of decomposition (Bai et al., 2021). Recent studies have emphasized that it is not merely the presence of decomposer taxa, but the functional traits and ecological strategies of microbial communities that determine decomposition trajectories (Chen et al., 2021b; Sveen et al., 2025). Bacterial decomposers are especially influential during early and intermediate stages of litter breakdown, where they mobilize labile carbon and nitrogen compounds, contribute to enzymatic diversity, and interact closely with changing litter chemistry (Buresova et al., 2021). Taxonomic and functional diversity among bacteria have been consistently linked to decomposition efficiency and stability, with more diverse communities exhibiting greater metabolic plasticity and resistance to disturbance (Błońska et al., 2021; Xue et al., 2025). In this context, microbial life-history strategies provide a powerful conceptual framework for interpreting how bacteria respond to environmental variability and substrate quality (Chen et al., 2021a; Zheng et al., 2021). Along the oligotroph–copiotroph continuum, oligotrophic bacteria are characterized by slow growth, high substrate-use efficiency, and tolerance to nutrient-poor or stressful conditions (Fierer et al., 2007; Yang et al., 2025). In contrast, copiotrophic bacteria exhibit rapid growth and resource exploitation under nutrient-enriched conditions (Du et al., 2024). These divergent strategies influence community assembly, succession, and functional output during decomposition (Bourget et al., 2023). However, empirical evidence directly connecting shifts in bacterial life-history strategies with dynamic changes in litter quality—particularly in stoichiometric parameters such as C:N:P ratios or lignin:TN—remains scarce.

The intrinsic chemical composition of plant litter is a major determinant of decomposition dynamics, influencing both microbial colonization and enzymatic activity (Cai et al., 2021). High lignin or lignin:N ratios are often associated with recalcitrant litter types that decompose slowly due to limited microbial degradability and enzyme inhibition, whereas litter with low C:N or C:P ratios and higher proportions of labile compounds tends to support faster microbial turnover (Luo et al., 2025). These chemical traits not only determine the energy and nutrient yields for microbial decomposers, but also shape the assembly and succession of microbial communities through stoichiometric constraints (Schroeder et al., 2020). Importantly, litter chemistry is not static during decomposition. As labile substrates are rapidly consumed, the relative proportion of recalcitrant materials increases, altering the resource landscape for microbes and imposing new selective pressures on community composition and functional traits. Bacterial taxa differ in their enzymatic capabilities and elemental requirements, making them differentially responsive to these temporal shifts in litter quality (Barbaccia et al., 2022). However, the degree to which bacterial community structure and life-history strategies track or respond to these chemical transformations remains unclear, particularly under conditions where environmental stress may override substrate-driven effects (Berg et al., 2022). Understanding these interactions is essential for disentangling the co-regulation of decomposition by litter traits and microbial ecology.

Dry-hot valley ecosystems, such as those found in southwest China, offer a valuable yet understudied context for examining the interplay between litter chemistry and microbial decomposition (Jin et al., 2024). These regions are characterized by intense solar radiation, high evapotranspiration rates, strong seasonal drought, and shallow, nutrient-poor soils (Mu et al., 2015). Such conditions impose multiple abiotic constraints on microbial activity, including thermal stress, desiccation, and nutrient limitation. Despite these challenges, herbaceous vegetation remains the dominant form of primary productivity, contributing substantial litter inputs to the soil (Feng et al., 2024). However, the decomposition of herbaceous litter under these stressful environmental conditions may follow fundamentally different trajectories than in more temperate or humid systems, particularly in terms of microbial succession and trait expression. In such environments, microbial communities are likely shaped by both resource constraints imposed by litter chemistry and physiological stresses from the surrounding environment (Babur et al., 2022). As a result, shifts in bacterial diversity, functional composition, and life-history strategies may occur more rapidly and be more tightly coupled to substrate availability. Yet, studies in dry-hot valley ecosystems rarely integrate microbial trait-based frameworks with litter quality assessments over time. This limits our ability to generalize decomposition models to arid and semi-arid environments, which are expanding globally under climate change.

In this study, we examined the decomposition dynamics of six dominant herbaceous plant species in a dry-hot valley ecosystem, with a specific focus on the bacterial community’s response to litter chemical changes over time. We conducted a 493-day litterbag experiment with five sequential sampling points, during which we assessed litter mass loss, quantified key chemical and stoichiometric traits (TC, TN, TP, cellulose, lignin, and derived stoichiometric ratios), and characterized bacterial communities using 16S rRNA gene sequencing at three representative decomposition stages (T1_69, T3_271, and T5_493). Litter mass loss and chemical traits were measured at five time points (T1_69–T5_493). We employed trait-based and statistical modeling approaches—including bacterial life-history strategy classification, random forest analysis, Pearson correlation, and canonical correspondence analysis (CCA)—to disentangle the relationships among bacterial traits, litter quality, and decomposition rate. Our specific objectives were to: (1) quantify temporal changes in litter decomposition and chemical composition; (2) track shifts in bacterial diversity, community composition, and life-history strategies across decomposition stages; (3) identify bacterial biomarkers associated with specific litter traits; and (4) determine the relative contributions of microbial and chemical factors in explaining decomposition variability. By integrating microbial functional traits with litter stoichiometry across a decomposition chronosequence, this study provides new insights into the ecological strategies of bacteria under environmental stress and contributes to a more mechanistic understanding of litter turnover in water-limited ecosystems.

## 2 Materials and methods

### 2.1 Site description and experimental design

This study focused on the decomposition of typical herbaceous plant litter in the dry-hot valley region of Yunnan Province, China. The litter samples were collected in January 2022 from the Xiaojian Mountain Experimental Base of the State-owned Forest Farm in Yongren County, located in the central part of the dry-hot valley area. This sampling time was chosen because January generally falls within the dry season in this region, when aboveground growth of most herbaceous plants has largely ceased and litter inputs are relatively stable, thereby minimizing potential variability in initial litter quality. This region is characterized by its unique climatic conditions, including high temperatures, seasonal drought, and low precipitation, typical of dry-hot valley ecosystems (Mu et al., 2015; Jin et al., 2024).

The plant species selected for the experiment included both perennial herbaceous plants (*Aristida adscensionis*, *Chloris virgata*, *Arthraxon prionodes*, and *Cymbopogon distans*) and annual herbaceous plants (*Urochioa longifolia* and *Dactyloctenium aegyptium*). These species were chosen based on their prevalence and ecological significance in the region. Litter samples of approximately 100 g from each species were collected in January 2022 and shade-dried in a well-ventilated indoor area for approximately 15 days until constant weight. After drying, 100 g of litter from each species was weighed and placed into each litterbag. Before burial, surface litter and loose debris were carefully removed to expose the mineral soil, and the litterbags were then buried at a depth of approximately 5 cm. For each species, a total of 20 litterbags were prepared, corresponding to four independent replicate litterbags for each of the five sampling time points. The decomposition process was monitored over a 493-day period, with litter samples collected at five time points: 69 days (T1_69), 138 days (T2_138), 271 days (T3_271), 369 days (T4_369), and 493 days (T5_493). At each collection point, litter mass loss was measured by weighing the litter samples before and after drying at 60°C for 48 h. In addition, litter chemical properties, including total carbon (TC), total nitrogen (TN), total phosphorus (TP), cellulose, and lignin, were analyzed at each time point to examine the changes in litter quality during decomposition. To investigate the microbial communities involved in decomposition, DNA was extracted from litter samples collected at three representative decomposition stages (T1_69, T3_271, and T5_493), corresponding to the early, intermediate, and late phases of decomposition.

### 2.2 Litter chemical properties analysis

Litter chemical properties were analyzed at five distinct decomposition stages: T1_69 (69 days), T2_138 (138 days), T3_271 (271 days), T4_369 (369 days), and T5_493 (493 days). Litter samples were dried at 60°C for 48 h before being ground to a fine powder. Total carbon (TC), total nitrogen (TN), and total phosphorus (TP) were measured using an elemental analyzer (Vario EL Cube, Elementar, Germany). Cellulose content was determined using the method described by Van Soest et al., 1991, and lignin content was measured by acid detergent fiber (ADF) method (Iiyama and Wallis, 1988). Lignin-to-nitrogen ratio (Lignin/TN) and other stoichiometric ratios such as TC:TN, TC:TP, and TN:TP were calculated based on the chemical properties of the litter at each sampling point. All chemical analyses were conducted in triplicate, and the data were used to evaluate changes in litter composition during decomposition and to examine correlations with microbial community dynamics.

### 2.3 High-throughput gene sequencing

Microbial DNA was extracted from approximately 0.20 g of frozen soil (–80°C) using PowerSoil DNA Isolation Kits (MoBio Laboratories, Carlsbad, CA, United States). The 338F (5’-ACTCCTACGGGAGGCAGCAG-3’) and 806R (5’-GGACTACHVGGGTWTCTAAT-3’) primers were using to amplify V3–V4 region of the bacterial 16S rRNA gene, sequencing was performed using the Illumina platform (Illumina, San Diego, United States). Raw sequences (> 300 bp length with an average quality score > 30) were analyzed using the Quantitative Insights Into Microbial Ecology pipeline (Caporaso et al., 2010). Amplified sequence variant (ASV) was clustered at 99% sequencesimilarity using UPARSE. The sequences were assigned to ASV with req-reference to a subset of the SILVA 128 database for bacteria (Edgar et al., 2011). The raw sequence data have been deposited in NCBI under the accession number (PRJNA90103) that are publicly accessible at www.ncbi.nlm.nih.gov

We defined Alphaproteobacteria, Gammaproteobacteria, Bacteroidota, and Firmicutes as copiotrophs strategy bacteria, Actinobacteriota, Verrucomicrobiota, Acidobacteriota, Gemmatimonadota, Chloroflexi, Planctomycetota as copiotrophs bacteria.

### 2.4 Statistical analysis

One-way analysis of variance (ANOVA) was used to assess differences in litter mass loss and chemical properties across the different decomposition stages. *Post hoc* Tukey’s HSD test was applied to identify significant differences between individual sampling stages. Pearson correlation analysis was conducted to determine relationships between bacterial diversity indices (Shannon and Chao1), life-history strategies, and litter chemical properties (e.g., C:N ratio, lignin content). Bacterial community composition and diversity patterns were assessed using principal coordinate analysis (PCoA) based on Bray-Curtis dissimilarity. Hierarchical partitioning was performed to assess the relative contributions of bacterial traits and chemical properties to the variation in litter mass loss rate, with significance determined using a permutation test (999 permutations). Random forest analysis was conducted to identify bacterial biomarkers associated with litter quality and decomposition stages, and the importance of environmental variables (e.g., lignin, cellulose) in explaining bacterial community structure was evaluated through canonical correspondence analysis (CCA). Statistical significance for all tests was considered at *P* < 0.05.

## 3 Results

### 3.1 Litter decomposition and chemical properties in different stages

**FIGURE 1:**
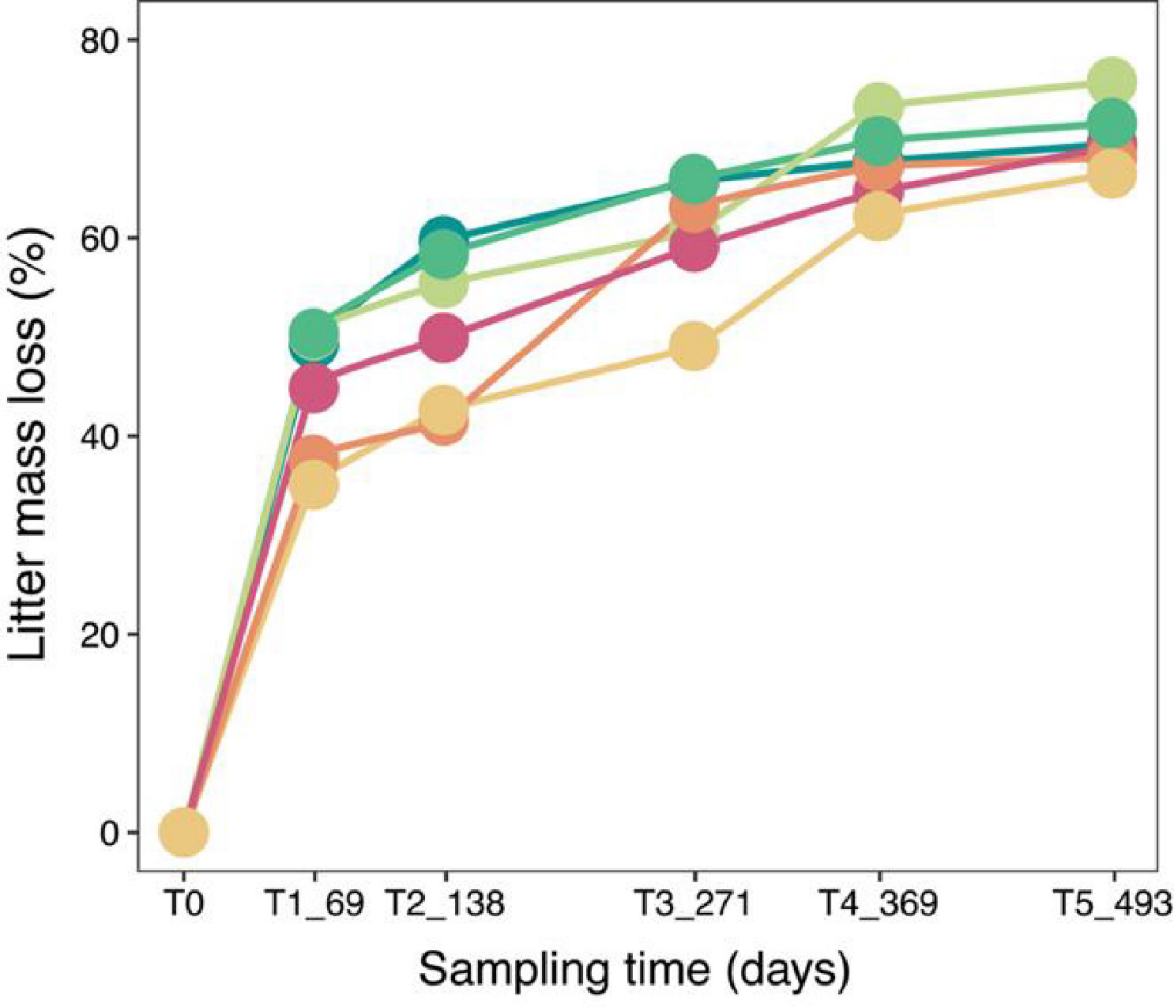
Litter mass loss percentage and mass loss rate of different plant species during decomposition. Percentage of litter mass loss at five decomposition stages (T1_69, T2_138, T3_271, T4_369, and T5_493). SMC, *Aristida adscensionis*; HWC, *Chloris virgata*; MYC, *Arthraxon prionodes*; YXC, *Cymbopogon distans*; WFC, *Urochloa longifolia*; LZC, *Dactyloctenium aegyptium*.

All six representative herbaceous species experienced significant litter mass loss over the 493-day decomposition period (Figure 1). Overall, decomposition followed a typical “fast–slow” pattern, with a rapid increase in mass loss during the early stage (T1_69), followed by a gradual plateau. By T5_493, the cumulative litter mass loss exceeded 70% for all species, and interspecific differences were no longer apparent, suggesting that decomposition duration was the primary driver of mass loss, whereas the influence of initial litter physicochemical properties appeared minimal. Consistent with these patterns, the exponential decay parameters further revealed differences in decomposition dynamics among species (Table 1). The decomposition constant (k) ranged from 0.728 to 0.910 yr^–1^, with MYC showing the highest k value (0.910 yr^–1^) and HWC showing the lowest (0.728 yr^–1^). Accordingly, the estimated time to reach 50% mass loss (T50%) varied from 278.1 to 347.7 days, while the time to reach 95% mass loss (T95%) ranged from 1201.9 to 1502.9 days (Table 1).

**TABLE 1:** Decomposition parameters of six herbaceous plant species.

Species	k (yr^–1^)	R	T50% (days)	T95% (days)
HWC	0.728	–0.842	347.7	1502.9
LZC	0.787	–0.872	321.3	1388.8
MYC	0.910	–0.923	278.1	1201.9
SMC	0.731	–0.957	346.3	1496.8
WFC	0.823	–0.938	307.4	1328.6
YXC	0.744	–0.916	340.2	1470.4

k, litter decomposition constant (yr^–1^); R, Pearson correlation coefficient; T50% and T95%, estimated time (days) to reach 50 and 95% litter mass loss, respectively.

Table 2 presents the changes in litter carbon, nitrogen, phosphorus, cellulose, and lignin contents along with their stoichiometric ratios across different decomposition stages. The results demonstrate that TC content was significantly higher in stages T3_271, T4_369, and T5_493 compared to T1_69 and T2_138 (*p* < 0.05), while TN and TP contents showed no significant differences among stages. The TC/TN and TC/TP ratios similarly exhibited no significant variations across stages. However, the TN/TP ratio was significantly elevated in stage T3_271 compared to T1_69, T2_138, T4_369, and T5_493 (*p* < 0.05). Cellulose content displayed a decreasing trend during decomposition, though inter-stage differences were not statistically significant. Lignin content was significantly higher in T4_369 than T3_271 (*p* < 0.05), and the Lignin/TN ratio followed the same pattern, being significantly greater in T4_369 than T3_271 (*p* < 0.05). These findings collectively indicate distinct temporal patterns in litter chemical properties during decomposition, with SOC accumulation and nutrient stoichiometry shifts being particularly notable.

**TABLE 2:** Changes in carbon, nitrogen, phosphorus, cellulose, lignin contents, and their stoichiometric ratios in litter across decomposition stages, averaged across six herbaceous plant species.

Properties	T1_69	T2_138	T3_271	T4_369	T5_493
SOC (g kg-^1^)	448.07 ± 7.82b	449.8 ± 7.7b	473.1 ± 5.94a	483.41 ± 6.57a	479.38 ± 5.98a
TN (g kg-^1^)	15.72 ± 0.75a	17.64 ± 2.67a	17.86 ± 2.78a	13.72 ± 1.52a	17.52 ± 1.76a
TP (g kg-^1^)	0.99 ± 0.05a	0.93 ± 0.13a	0.82 ± 0.15a	1.02 ± 0.12a	0.9 ± 0.11a
TC/TN	28.93 ± 1.81a	28.3 ± 3.84a	29.33 ± 3.8a	37.27 ± 3.85a	28.89 ± 3.12a
TC/TP	456.13 ± 18.94a	549.76 ± 105.82a	730.93 ± 172.73a	502.6 ± 53.65a	575.05 ± 75.09a
TN/TP	16.1 ± 1.24b	19.32 ± 1.69ab	24.3 ± 3.5a	14.23 ± 1.76b	19.79 ± 1.12ab
Cellulose (g kg-^1^)	107.56 ± 8.15a	95.24 ± 21.92a	92.99 ± 16.71a	81.95 ± 7.78a	78.54 ± 5.63a
Lignin (g kg-^1^)	180.01 ± 14.54ab	186.3 ± 19.63ab	164.62 ± 15.24b	227.77 ± 13.51a	208.64 ± 19.76ab
Lignin/TN	0.4 ± 0.03ab	0.41 ± 0.04ab	0.35 ± 0.03b	0.47 ± 0.02a	0.44 ± 0.04ab

Values are means ± SE across six herbaceous plant species at each decomposition stage. Different lowercase letters indicate significant differences (*p* < 0.05; two-way ANOVA; Tukey’s *post-hoc* test) between treatments.

### 3.2 Bacteria community diversity and life-history strategies

**FIGURE 2:**
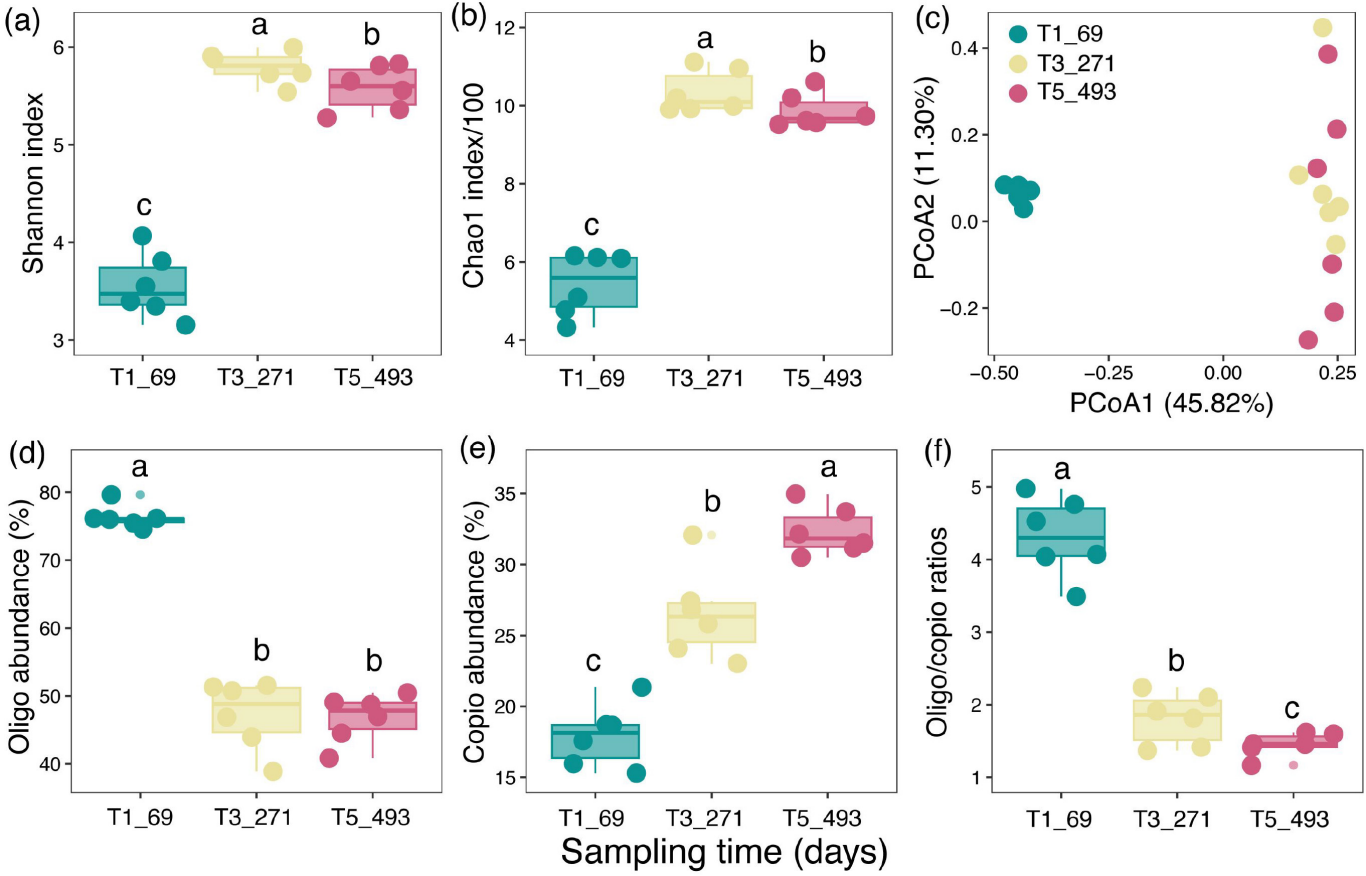
Changes in bacterial diversity, community composition, and life-history strategies during litter decomposition. **(a–f)** Samples are colored by sampling time: T1_69 (blue), T3_271 (yellow), and T5_493 (red). Different lowercase letters indicate significant differences (*p* < 0.05; two-way ANOVA; Tukey’s *post-hoc* test) between treatments.

The diversity of bacterial communities underwent significant changes during the decomposition process (Figure 2). The Shannon index and Chao1 index (Figures 2a,b) reached their highest values during the T3_271 stage, indicating an increase in bacterial diversity and richness at the intermediate decomposition stage. Principal coordinate analysis (PCoA) further confirmed this change, with samples from the T1_69 stage showing significant separation from those of T3_271 and T5_493 in the PCoA plot (Figure 2c), demonstrating distinct shifts in bacterial community composition over time. Bacterial life-history strategies also exhibited significant shifts as decomposition progressed (Figures 2d–f). In the early decomposition stage (T1_69), oligotrophic bacteria were relatively more abundant, while copiotrophic bacteria gradually became dominant over time. The Oligo/Copio ratio decreased as decomposition advanced, indicating a transition from communities dominated by resource-efficient, stress-tolerant oligotrophs to those dominated by fast-growing, resource-demanding copiotrophs. This shift reflects the changing ecological strategies of microbial communities across decomposition stages.

**FIGURE 3:**
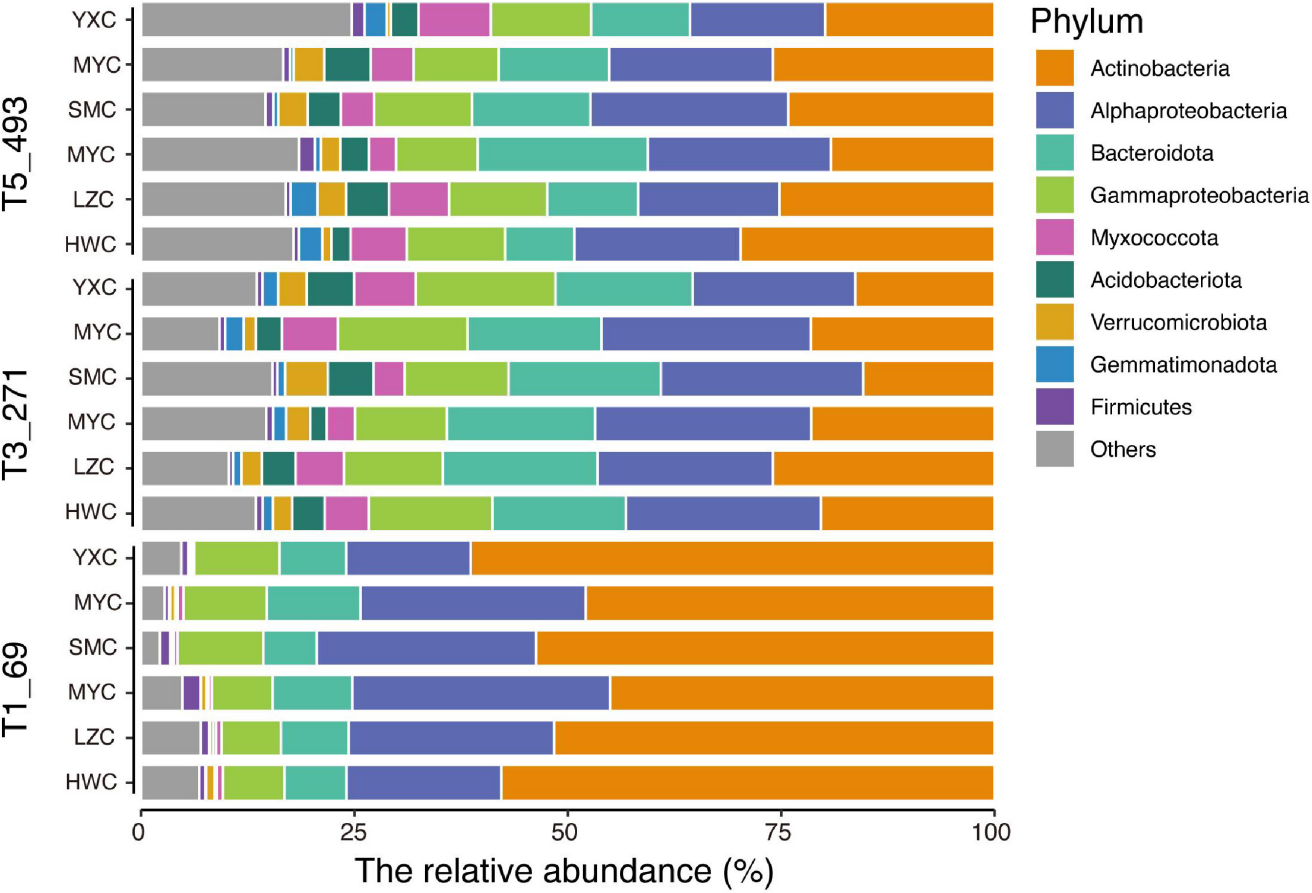
Changes in the relative abundance of bacterial phyla in litter of six herbaceous plant species across decomposition stages.

During decomposition, the composition of bacterial communities at the phylum level varied significantly among the six herbaceous plant species (Figure 3). At the T1_69 stage, Actinobacteriota dominated the bacterial communities. As decomposition progressed, Bacteroidota became increasingly abundant during the T3_271 and T5_493 stages. The bacterial community structure also varied among plant species. In the bar plots, Aristida (SMC) and Arthraxon (MYC) showed higher relative abundances of Proteobacteria and Bacteroidota in the early decomposition stages, whereas Dactyloctenium (LZC) exhibited higher Acidobacteriota abundance, particularly in the later stages (T3_271 and T5_493).

### 3.3 Relationship between bacteria community and litter chemical properties

**FIGURE 4:**
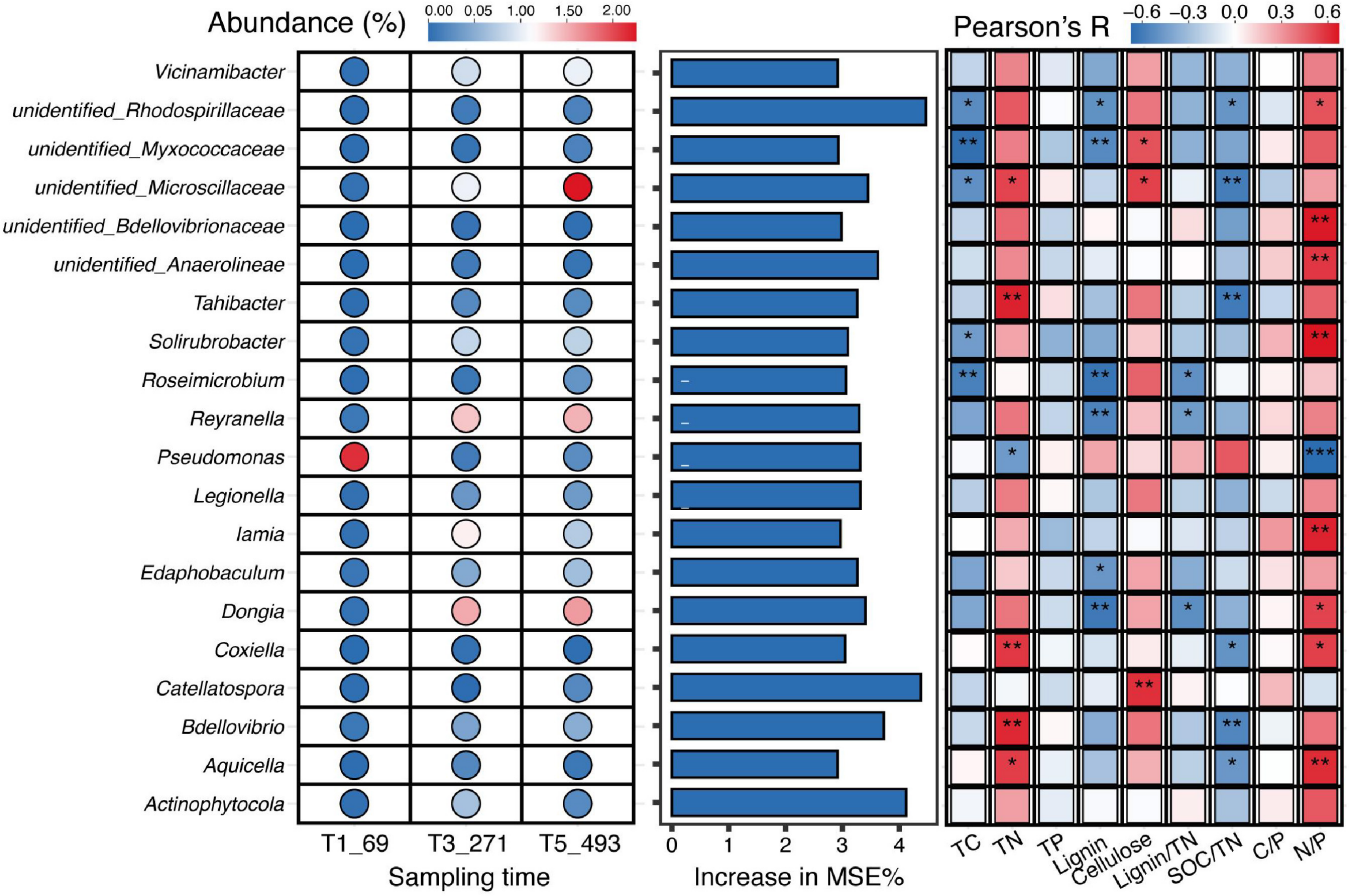
Identification of bacterial biomarkers at genera level (top 20) identified by random forest and their relatively abundance, as well as Pearson correlations between the top 20 bacterial genera (biomarkers identified by random forest) and environmental variables during litter decomposition. Significance levels are indicated by asterisks (*p* < 0.05, *p* < 0.01, *p* < 0.001).

Through random forest analysis, we identified 20 bacterial genera as biomarkers, whose relative abundances showed significant variations across different decomposition stages (Figure 4). These biomarkers included *Actinophytocola*, *Pseudomonas*, *Coxiella*, and *Legionella*, demonstrating distinct abundance patterns during the decomposition of various plant litter types. As decomposition progressed (T1_69, T3_271, T5_493), the relative abundances of these bacterial genera underwent significant temporal changes, exhibiting clear stage-dependent dynamics. Pearson correlation analysis further revealed relationships between the abundance of these bacterial genera and multiple environmental factors (e.g., TC, TN, lignin content, cellulose content, Lignin/TN ratio, etc.) (Figure 4). For instance, the abundance of *Pseudomonas* showed a significant positive correlation with cellulose content, while *Actinophytocola* abundance was negatively correlated with TN content.

**FIGURE 5:**
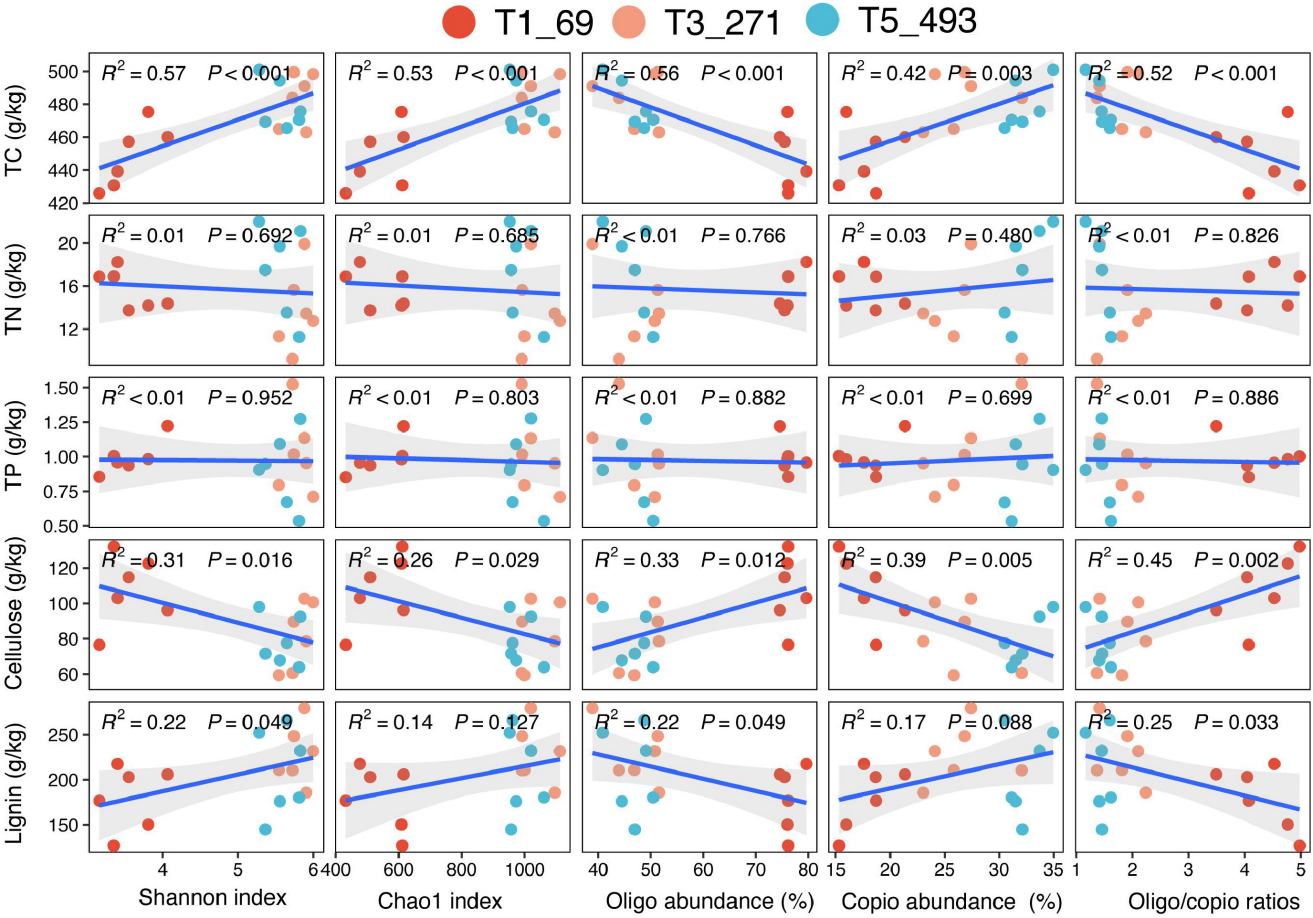
Correlations between bacterial community alpha diversity and life-history strategies and litter physicochemical properties, including total carbon (TC), total nitrogen (TN), total phosphorus (TP), cellulose, and lignin.

The relationship between bacterial community characteristics and litter properties revealed significant patterns (Figure 5). The Shannon diversity index, Chao1 index, and the abundance of copiotrophic microbial groups showed significant positive correlations with TC and lignin content, while exhibiting significant negative correlations with cellulose. Conversely, the abundance of oligotrophic microbial groups and the ratio of oligotrophic to copiotrophic microbial groups demonstrated significant negative correlations with TC and lignin content but significant positive correlations with cellulose. However, no significant relationships were observed between bacterial life-history strategies and diversity with litter TN, TP, C/N, C/P, N/P, or Lignin/TN (Figure 5 and Supplementary Figure 1).

**FIGURE 6:**
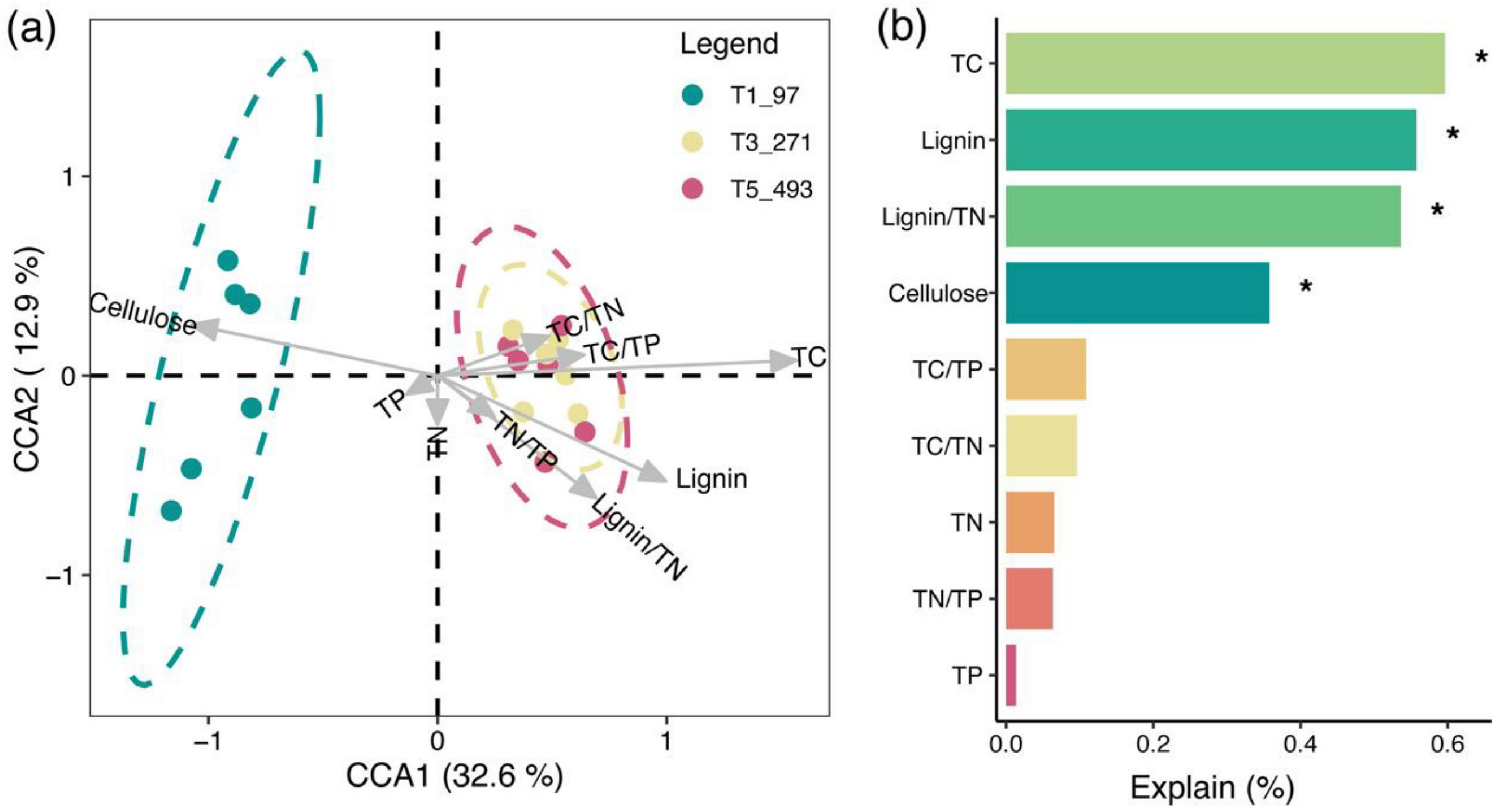
Effects of litter physicochemical properties on bacterial community composition. **(a)** Canonical Correspondence Analysis (CCA) shows the association between bacterial communities and environmental variables across decomposition stages. Arrows indicate environmental gradients; ellipses represent 95% confidence intervals. **(b)** Variable importance in explaining community variation, with asterisks denoting significant effects (*p* < 0.05).

Canonical Correspondence Analysis (CCA) revealed the associations between bacterial communities and environmental variables across different decomposition stages (Figure 6). Arrows indicate environmental gradients, and ellipses represent 95% confidence intervals. The results demonstrate that environmental variables such as TC, TN, cellulose content, and Lignin/TN significantly influence the composition of bacterial communities, with distinct distributions observed at different decomposition stages. Variable importance analysis illustrates the contribution of each environmental variable in explaining the variation in bacterial communities, with significant variables (*p* < 0.05) marked by asterisks. TC and cellulose content play crucial roles in driving bacterial community changes, particularly across different decomposition stages, highlighting their selective influence on bacterial community composition.

### 3.4 Dominant controls of litter decomposition rate

**FIGURE 7:**
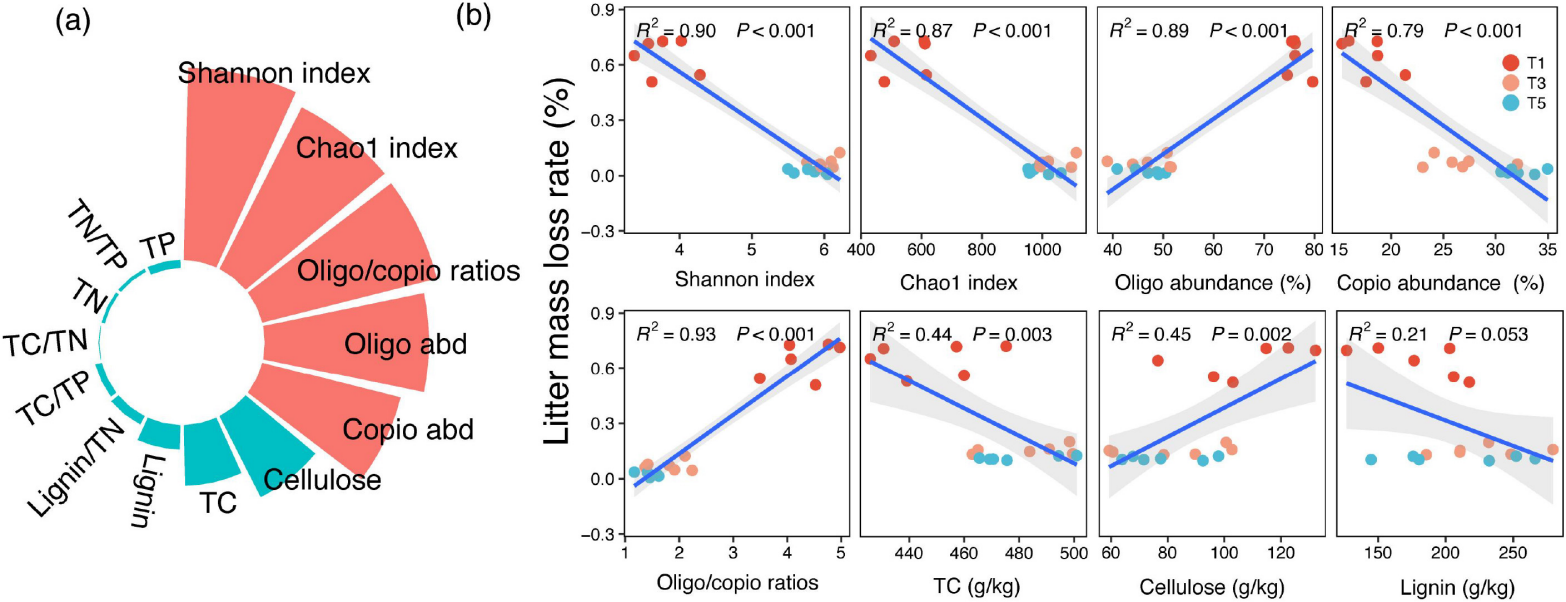
**(a)** Results of hierarchical partitioning analysis illustrating the independent contributions of various factors to litter mass loss rate variation. The *R*^2^ value reflects the proportion of variance in litter mass loss rate explained by the model. **(b)** Correlation analysis between litter mass loss rate and bacterial community alpha diversity and life-history strategies and litter physicochemical properties.

The results of hierarchical partitioning analysis revealed that bacterial traits accounted for a substantially larger proportion of the cumulative variance (48.61%) in litter mass loss rate compared to litter chemical properties (10.61%) (Figure 7a). Overall, litter mass loss rate exhibited positive correlations with the abundance of oligotrophic microbial groups, the ratio of oligotrophic to copiotrophic microbial groups and cellulose content. In contrast, SOC content showed a negative correlation with the Shannon index, Chao1 index, and the abundance of copiotrophic microbial groups, as well as TC and lignin content (Figure 7b). However, litter mass loss rate showed no significant relationships with TN, TP, TC/TN, TC/TP, TN/TP, or Lignin/TN (Supplementary Figure 2).

## 4 Discussion

In this study, we observed that litter decomposition followed a clear temporal pattern, with an initial rapid breakdown followed by a stabilization phase. The majority of the mass loss occurred during the early stages (T1_69 and T2_138), with decomposition rates slowing as time progressed. By the later stages of decomposition (T3_271, T4_369, and T5_493), the mass loss rates of all six herbaceous species had significantly reduced, and the decomposition process appeared to stabilize. This pattern aligns with the typical “fast–slow” decomposition model (Bradford et al., 2016), where microbial activity and the breakdown of labile organic matter are most rapid initially, while more recalcitrant compounds, such as lignin and cellulose, take longer to degrade (Berg et al., 2022; Dong et al., 2025). Consistent with the temporal “fast–slow” pattern, the exponential decay parameters further quantified interspecific differences in decomposition dynamics. The decomposition constant ranged from 0.728 to 0.910 yr^–1^, with MYC showing the highest decay rate and HWC the lowest. Such variation is consistent with the notion that initial litter quality and its stage-dependent changes can modulate the accessibility of substrates to decomposers, thereby affecting the apparent decay constant during the early-to-intermediate decomposition period, even when long-term cumulative mass loss eventually becomes less distinguishable among species (Neiske et al., 2025). Importantly, while differences in litter chemistry (e.g., lignin content and C:N ratios) existed across species, the overall trajectory of decomposition remained consistent. All species showed rapid initial breakdown followed by a slowing down of decomposition rates. Overall, this supports a strong time (stage) effect on decomposition dynamics, whereas the influence of N- and P-related variables appeared comparatively weaker in our dataset. In dry-hot valley ecosystems, early stage decomposition may be shaped not only by substrate availability but also by microenvironmental constraints (e.g., moisture limitation), which can influence the realized microbial strategies operating on litter substrates (Berg et al., 2022; Zhao et al., 2025). These findings imply that microbial communities initially take advantage of more labile carbon substrates, and as these are depleted, the decomposition process slows due to the increasing dominance of recalcitrant compounds like lignin and cellulose.

Microbial diversity and life-history strategies play a crucial role in driving the decomposition of organic matter, particularly in environments with limited resources (Yang et al., 2023). For bacterial community analyses, we sequenced three strategically chosen time points (T1_69, T3_271, and T5_493) because they capture the major phase transitions of the decomposition trajectory (early rapid loss, intermediate transition, and late stabilization), providing a concise representation of bacterial community turnover along the decomposition gradient. Bacterial alpha diversity exhibited a clear temporal pattern across decomposition stages, with both Shannon and Chao1 peaking at the intermediate stage (T3_271) and showing lower values at the early stage (T1_69) and the late stage (T5_493). This mid-stage maximum suggests that, as decomposition proceeds and litter substrates diversify in accessibility, a broader set of bacterial taxa can coexist before the community becomes more selective again under late-stage resource limitation and increasing substrate recalcitrance (Lin et al., 2025). In our study, the early stages of decomposition, dominated by rapidly degrading labile carbon sources, showed lower diversity, likely due to the selective advantage of specific taxa capable of utilizing these compounds efficiently (Li et al., 2021). As decomposition progressed, bacterial diversity increased, particularly in the middle stages (T3_271), when more diverse microbial communities became involved in the degradation of complex and recalcitrant materials such as lignin and cellulose. This pattern suggests that as the easier-to-degrade organic matter was consumed, the microbial community expanded to include a wider range of taxa adapted to breaking down more challenging substrates (Banerjee et al., 2018; Bourget et al., 2023). In the later stages (T5_493), the diversity decreased again as more recalcitrant substrates persisted and decomposition slowed, leading to a reduction in the diversity of functional groups actively involved in the process.

The dynamics of microbial life-history strategies are central to understanding how bacterial communities influence decomposition rates (Chen et al., 2021a). In this study, we observed significant shifts in bacterial life-history strategies throughout the decomposition process, particularly in relation to the changing availability of carbon substrates (Babur et al., 2022). Early in decomposition (T1_69), oligotrophic (slow-growing) bacteria were relatively more abundant. These bacteria are typically adapted to nutrient-limited environments and are more efficient in degrading complex organic compounds such as lignin and cellulose, which are abundant in litter during the early stages of decomposition. This initial dominance of oligotrophs suggests that their ability to efficiently utilize available, albeit more recalcitrant, substrates give them a competitive advantage in the nutrient-poor conditions created by the breakdown of labile carbon sources (Lauro et al., 2009; Hu et al., 2024).

Consistent with the observed shifts in community structure, bacterial life-history strategies changed markedly with decomposition. Oligotrophic taxa were relatively more abundant at the early stage (T1_69), whereas copiotrophic taxa gradually became dominant toward the later stage (T5_493), leading to a decreasing Oligo/Copio ratio (Figures 2d–f). This pattern indicates a transition from resource-efficient, stress-tolerant communities early on to communities characterized by faster-growing, resource-demanding taxa as decomposition advances (Fierer et al., 2007). Such stage-dependent shifts likely reflect microbial adjustment to changing litter chemistry and microenvironmental conditions during decomposition, whereby shifts in substrate availability and quality are accompanied by a reorganization of community life-history strategies. Interestingly, despite the increased dominance of copiotrophic bacteria during the mid-stages of decomposition, we observed that the abundance of oligotrophic bacteria remained relatively high in the later stages (T5_493). This indicates that, even in the presence of labile carbon substrates, oligotrophic bacteria continue to play a role in breaking down more complex and recalcitrant organic matter, such as lignin and cellulose, which dominate in the later stages of decomposition (Lohbeck et al., 2013; Huang et al., 2025). This persistence of oligotrophs may reflect the microbial community’s adaptive capacity to persist under nutrient-limited conditions and to continue processing more challenging substrates as decomposition progresses (Eichorst et al., 2018; Zimmerman et al., 2020).

Regarding the decomposition rate of litter, we observed that both microbial diversity and life-history strategies play significant roles in determining litter decomposition rates, alongside the chemical composition of the litter itself. The litter’s chemical traits, particularly lignin content, cellulose, and the C:N ratio, were critical in shaping microbial community composition and activity during the decomposition process (Lohbeck et al., 2013; Bourget et al., 2023). The chemical composition of the litter directly influenced microbial community structure and functional traits (Zheng et al., 2021). Linking community attributes to litter properties further supports these interpretations. Shannon and Chao1, together with copiotrophic groups, were positively associated with TC and lignin but negatively associated with cellulose, whereas oligotrophic groups showed the opposite pattern. Moreover, our variance-partitioning results indicate that bacterial traits explained more variation in litter mass loss than bulk litter chemical properties, and litter mass loss rate was positively associated with oligotrophic abundance and cellulose, but negatively associated with alpha diversity, copiotrophic abundance, TC, and lignin. Together, these results suggest that shifts in bacterial ecological strategies are closely coupled to substrate-quality gradients along the decomposition chronosequence and can outweigh bulk chemical metrics in explaining decomposition-rate variability (Waring et al., 2022).

The dominance of oligotrophic bacteria in the early stages of decomposition is particularly noteworthy, as it suggests that these bacteria are capable of efficiently utilizing the available recalcitrant carbon substrates, despite the presence of labile carbon (Schroeder et al., 2020; Su et al., 2023). Their persistence through various stages of decomposition points to their critical role in the breakdown of structurally complex materials like lignin and cellulose, which are key components of the litter in arid ecosystems (Zhao et al., 2025). While microbial diversity and life-history strategies explain much of the variance in decomposition rates, litter chemistry is equally important in determining the efficiency of decomposition (Shao et al., 2021; Bourget et al., 2023). The Lignin/TN was particularly influential in driving shifts in microbial community composition and function (Bennett et al., 2015). Litter with a high Lignin/TN ratio typically slows decomposition, as lignin is a complex and resistant compound. In contrast, lower Lignin/TN ratios are associated with faster decomposition rates, which is consistent with the increased dominance of copiotrophic bacteria in these conditions. Moreover, microbial diversity was positively correlated with litter carbon and nitrogen content, indicating that richer, more nutrient-dense litter types support more diverse microbial communities (Santonja et al., 2017; Luo et al., 2025). The presence of a wide range of microbial taxa likely enhances the efficiency of decomposition by promoting the breakdown of various components of the litter. The decreasing diversity at later stages of decomposition corresponds to a more specialized community of bacteria focused on the degradation of recalcitrant materials, further emphasizing the importance of microbial functional traits in the decomposition process (Buresova et al., 2021; Qi et al., 2023).

Despite the insights gained from this study, several limitations should be acknowledged. Our microbial assessment focused on bacterial communities, whereas fungi are important decomposers of structurally complex substrates (Ye et al., 2024). Future studies incorporating fungal community data would strengthen inference on multi-kingdom decomposer dynamics and potential cross-kingdom interactions (Zhao et al., 2025). In addition, decomposition was examined under field conditions to reflect natural processes, but microclimatic variability, especially moisture and temperature heterogeneity, may influence decomposition dynamics in dry-hot valley ecosystems (Li et al., 2025). Continuous microclimate monitoring and targeted moisture manipulations would help disentangle substrate-quality effects from abiotic controls. Finally, functional interpretation in this study was mainly inferred from taxonomic patterns and life-history strategy proxies. Integrating functional approaches such as metagenomics and enzyme assays would provide more direct evidence linking community shifts to lignocellulose-degrading potential and stress-response traits (Rodriguez del Rio et al., 2025).

## 5 Conclusion

This study highlights the significant role of bacterial communities in litter decomposition in a dry-hot valley ecosystem based on a 493-day litterbag experiment with six representative herbaceous species. We found that bacterial life-history strategies, including shifts from copiotrophic to oligotrophic taxa, are key drivers of decomposition, with bacterial traits accounting for a larger proportion of variance in litter mass loss than litter chemical properties. Overall, litter mass loss followed a clear “fast–slow” pattern, and species-specific decay parameters further indicated interspecific differences in decomposition rates. Bacterial diversity and community composition varied over time, influenced by changes in litter quality, particularly cellulose and lignin content. Our findings also identified key bacterial biomarkers linked to specific litter traits, such as *Pseudomonas* and *Actinophytocola*, indicating that trait-associated bacterial groups may track substrate quality changes during decomposition. The results underscore the importance of litter stoichiometry and microbial traits in shaping decomposition dynamics, providing valuable insights into microbial adaptation to environmental constraints in dryland ecosystems. This work advances our understanding of microbial mediation in litter turnover and offers implications for predicting decomposition in the face of climate change.

## Data Availability

The original contributions presented in this study are included in this article/Supplementary material, further inquiries can be directed to the corresponding author.
