# Supplementary material for: Linking bacterial life-history strategies and diversity to litter decomposition dynamics in a dry-hot valley area
Source: Front Microbiol. 2026 Feb 6;17:1766521. doi: 10.3389/fmicb.2026.1766521 (PMC12920599; doi:10.3389/fmicb.2026.1766521)
Supplement: Supplementary file 1 [file Supplementary_file_1.pdf]

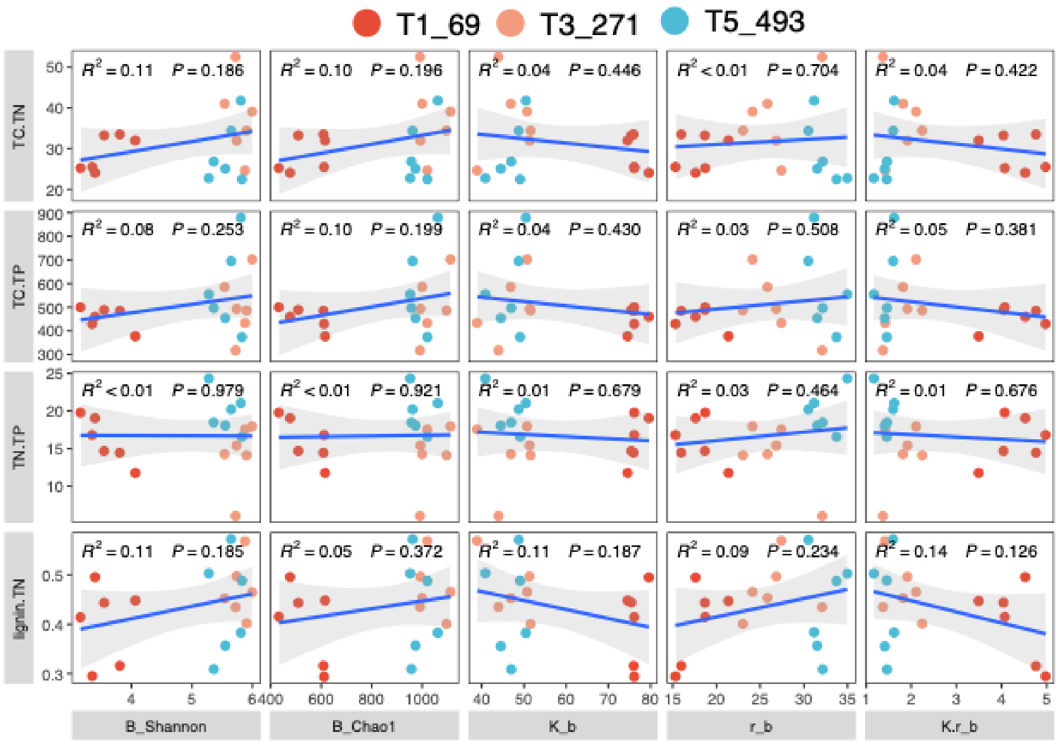

Figure S1. Correlations between bacterial community traits and litter stoichiometric ratios (C/N, C/P, N/P, and Lignin/TN). Samples are colored by sampling time: T1_69 (blue), T3_271 (yellow), and T5_493 (red).

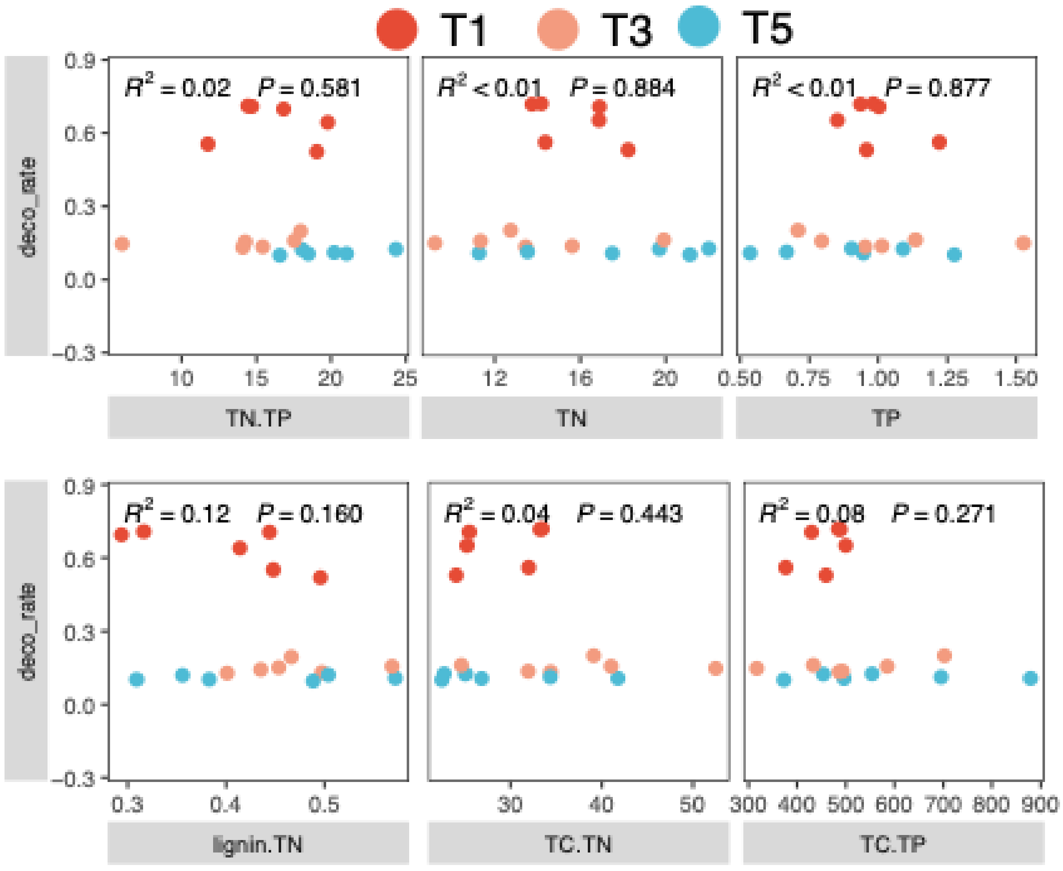

Figure S2. Correlation analysis between litter mass loss rate and litter physicochemical properties. Samples are colored by sampling time: T1_69 (blue), T3_271 (yellow), and T5_493 (red).
